# *Escherichia coli RimI* Encodes Serotonin *N*-Acetyltransferase Activity and Its Overexpression Leads to Enhanced Growth and Melatonin Biosynthesis

**DOI:** 10.3390/biom13060908

**Published:** 2023-05-30

**Authors:** Kyungjin Lee, Kyoungwhan Back

**Affiliations:** Department of Molecular Biotechnology, College of Agriculture and Life Sciences, Chonnam National University, Gwangju 61186, Republic of Korea; nicekj7@hanmail.net

**Keywords:** archaea, *Escherichia coli*, *N*-acetylserotonin, protein acetylation, RimI, melatonin, 5-methoxytryptamine

## Abstract

Serotonin *N*-acetyltransferase (SNAT) functions as the penultimate or final enzyme in melatonin biosynthesis, depending on the substrate. The *Escherichia coli* orthologue of archaeal SNAT from *Thermoplasma volcanium* was identified as RimI (EcRimI), with 42% amino acid similarity to archaeal SNAT. EcRimI has been reported to be an *N*-acetyltransferase enzyme. Here, we investigated whether EcRimI also exhibits SNAT enzyme activity. To achieve this goal, we purified recombinant EcRimI and examined its SNAT enzyme kinetics. As expected, EcRimI showed SNAT activity toward various amine substrates including serotonin and 5-methoxytryptamine, with *K*_m_ and *V*_max_ values of 531 μM and 528 pmol/min/mg protein toward serotonin and 201 μM and 587 pmol/min/mg protein toward 5-methoxytryptamine, respectively. In contrast to the *rimI* mutant *E. coli* strain that showed no growth defect, the *EcRimI* overexpression strain exhibited a 2-fold higher growth rate than the control strain after 24 h incubation in nutrient-rich medium. The *EcRimI* overexpression strain produced more melatonin than the control strain in the presence of 5-methoxytryptamine. The enhanced growth effect of *EcRimI* overexpression was also observed under cadmium stress. The higher growth rate associated with *EcRimI* expression was attributed to increased protein *N*-acetyltransferase activity, increased synthesis of melatonin, or the combined effects of both.

## 1. Introduction

Melatonin is a universal molecule present in almost all living organisms, including bacteria, archaea, plants, and animals [1,2,3]. Its primary identified function is associated with its potent antioxidant activity, as one molecule of melatonin can scavenge free radicals in a reaction cascade consuming up to 10 free radicals [4,5]. In addition to its intrinsic antioxidant activity, melatonin plays an important biological role in a number of species-specific functions. For example, it is a well-known pineal hormone that regulates the circadian rhythm and seasonal reproduction in animals [6,7]. By contrast, in plants, it acts as a master regulator of growth and development, affecting seed germination [8,9], photomorphogenesis [10], flowering [11,12], senescence [13], and grain yield [14,15] in concert with numerous plant hormones [16,17]. It also plays a pivotal role in alleviating plant damage resulting from various environment stresses, including abiotic and biotic stresses, either through enhancement of antioxidant activities and various defense genes [18,19] or improvement of protein quality control [12,20]. Melatonin is synthesized by bacteria, and appears to be involved in detoxifying reactive oxygen species to prevent free radical attack [21,22,23], although it has also been reported to inhibit the growth of plant-pathogenic bacteria [24].

In melatonin biosynthesis, the aromatic amino acid tryptophan serves as the initial substrate and melatonin is synthesized via four enzymatic reactions with tryptophan 5-hydroxylase (TPH), aromatic amino acid decarboxylase (tryptophan decarboxylase), serotonin *N*-acetyltransferase (SNAT; also named arylakylamine *N*-acetyltransferase), and *N*-acetylserotonin *O*-methyltransferase (ASMT) in animals. Analogously, plants also employ four enzymes, but TPH is replaced with tryptamine 5-hydroxylase, which catalyzes tryptamine into serotonin [25]. Among these four enzymes, SNAT plays a key role in melatonin biosynthesis, functioning as either the penultimate or final enzyme of melatonin biosynthesis, depending on substrate, in both animals and plants [2,25]. SNAT acetylates serotonin and 5-methoxytryptamine into *N*-acetylserotonin and melatonin, respectively [26]. In 1995, the first *SNAT* gene was cloned from sheep using a cDNA expression library [27], and its orthologues have been cloned and characterized from a wide range of species including Gram-positive bacteria and fungi but not from higher plants, nematodes, or arthropods [28]. Later, the first plant *SNAT* gene was cloned from the rice GCN5-related *N*-acetyltransferase (GNAT) family [29]. As expected, the SNAT protein from rice did not exhibit sequence homology with that from animals. The possible ancestor of the plant *SNAT* gene has been cloned and characterized in cyanobacteria [30].

Despite reports of *SNAT* genes from a diverse array of species, the presence of *SNAT* in archaea has long remained a mystery. Surprisingly, the archaeal *SNAT* gene from *Thermoplasma volcanium* was recently cloned [3] and a human orthologue was discovered [31]. This archaeal SNAT also belongs to the GNAT family, but it is classified as a new clade of SNAT, distinct from those of animals and plants. In particular, the Gram-negative bacterium *Escherichia coli* had long been reported to produce melatonin [32], but no *SNAT* homolog genes have yet been described. However, based on the discovery of archaeal *SNAT*, we identified an orthologous gene in *E*. *coli*.

In this study, we selected *E*. *coli RimI* (EcRimI), an archaeal *SNAT* orthologue expressed in *E*. *coli*, and purified recombinant EcRimI. We found that EcRimI possessed SNAT enzyme activity. Furthermore, its overexpression of *EcRimI* in *E*. *coli* was functionally linked to enhanced melatonin biosynthesis in the presence of 5-methoxytryptamine. The *EcRimI* overexpression strain of *E*. *coli* exhibited enhanced growth at 28 and 37 °C compared to the control strain. We concluded that enhanced synthesis of melatonin in the *EcRimI* overexpression strain was in part responsible for the enhanced growth rate due to ameliorating of starvation and stationary-phase stress, although we cannot rule out a possible role of protein acetylation.

## 2. Materials and Methods

### 2.1. Synthesis of Escherichia coli RimI Gene

Based on the nucleotide sequence information of *E. coli RimI* (GenBank accession number WP_137442509), the full-length *EcRimI* gene with the length of 447 bp was custom-synthesized at Bioneer (Daejeon, Republic of Korea).

### 2.2. Escherichia coli Expression and Purification of Recombinant EcRimI Protein

The full-length synthetic *EcRimI* gene was amplified by PCR using primer set (*RimI* forward primer, 5′-GCC ATG GGA AAC ACG ATT TCT TCC CTC GAA-3′; *RimI* reverse primer, 5′-CTC GAG CAT ACT GAT TGG CAA CGC-3′) with a template plasmid containing *EcRimI* DNA such as pBHA-RimI, which was provided by Bioneer. The PCR product was ligated into the TA vector (RBC Bioscience, New Taipei City, Taiwan) followed by *Nco*I and *Xho*I digestion. The *Nco*I and *Xho*I insert of *EcRimI* was then ligated into the same restriction sites of the *E. coli* expression vector pET28b (Invitrogen, Carlsbad, CA, USA), leading to the generation of pET28b-RimI. The pET28b-RimI plasmid was transformed into *E. coli* strain BL21(DE3) (Invitrogen). An overnight culture (10 mL) grown in Lennox broth (LB) medium (10 g/L pancreatic digest of casein, 5 g/L yeast extract, and 5 g/L NaCl) containing antibiotic kanamycin (50 mg/L) was inoculated into 100 mL of Terrific broth (TB) medium [20 g/L Bacto-tryptone, 24 g/L Bacto-yeast extract, glycerol 4 mL/L, and phosphate buffer (0.017 M KH_2_PO_4_ and 0.072 M K_2_HPO_4_)] containing 50 mg/L kanamycin and incubated at 37 °C for 4 h, followed by the addition of 1 mM isopropyl-β-D-thiogalactopyranoside (IPTG; Sigma, St. Louis, MO, USA). The culture was further grown at 24 °C and shaken at 180 rpm for 12 h. The purification procedure using affinity (Ni^2+^) chromatography was performed according to the manufacturer’s recommendations (Qiagen, Tokyo, Japan).

### 2.3. Homology Analysis

The analysis of amino acid sequence homology was carried out using the BLASTp tool in the non-redundant protein sequences databases at the National Center for Biotechnology Information (http://www.ncbi.nlm.nih.gov/, accessed on 3 September 2019). Phylogenetic tree analysis was performed using the BLAST-Explorer program [33].

### 2.4. Measurement of Serotonin N-Acetyltransferase Enzyme Kinetics

The purified recombinant EcRimI protein (3 μg or varying concentrations) was incubated in a total volume of 100 µL containing 0.5 mM serotonin (or other substrates) and 0.5 mM acetyl-CoA in 100 mM potassium phosphate (pH 8.8 or varying pH) at 55 °C (or other temperatures) for 30 min. Enzymatic reaction products such as *N*-acetylserotonin, *N*-acetyltryptamine, *N*-acetyltyramine, and melatonin were subjected to high-performance liquid chromatography (HPLC) analysis as described previously [11]. Lineweaver–Burk plots were employed to calculate substrate affinity (*K*_m_) and the maximum reaction rate (*V*_max_) using two substrates such as serotonin and 5-methoxytryptamine. Protein concentrations were determined using the Bradford method and a protein assay dye (Bio-Rad, Hercules, CA, USA). The analysis was performed in triplicate.

### 2.5. Growth Measurement of Escherichia coli

A 1 mL seed culture that had been incubated overnight at 37 °C in Lennox broth (LB) medium (10 g/L pancreatic digest of casein, 5 g/L yeast extract, 5 g/L NaCl) containing 50 mg/L kanamycin was inoculated into 10 mL Terrific broth (TB) medium containing 50 mg/L kanamycin in 40 mL polypropylene conical tubes (SPL Life Science, Pocheon-si, Republic of Korea) and continuously cultured at various temperatures. The absorbance at 600 nm of the culture was measured using a spectrophotometer (MicroDigital Nabi, GyungGi, Republic of Korea) periodically until 24 h.

### 2.6. Melatonin Measurement in Escherichia coli

One hundred microliters of seed culture incubated overnight at 37 °C in LB medium containing 50 mg/L kanamycin was inoculated into 1 mL LB medium containing 50 mg/L kanamycin and incubated at 37 °C until the optical density of the *E*. *coli* culture at 600 nm (OD600) reached 1.0. After the addition of 1 mM isopropyl-β-D-thiogalactopyranoside and 1 mM 5-methoxytryptamine, the culture was grown and shaken at 250 rpm at varying temperatures such as 28, 37, and 42 °C for the indicated time periods. The resulting cultures were centrifuged at 11,500× *g* for 5 min and separated into cell pellet and medium (supernatant) fractions. The medium fractions (0.2 mL) were mixed with 0.2 mL of 100% methanol. The resulting 10 µL aliquots were subjected to high-performance liquid chromatography (HPLC) using a fluorescence detector system (Waters, Milford, MA, USA) as described previously [34].

### 2.7. Cadmium Treatment of Escherichia coli

One hundred microliters of seed culture incubated overnight at 37 °C in LB medium containing 50 mg/L kanamycin was inoculated into 1 mL LB medium containing 50 mg/L kanamycin and incubated at 37 °C until the optical density of the *E*. *coli* culture at 600 nm (OD_600_) reached either 0.5 or 1.0. After adding 1 or 5 mM cadmium chloride (CdCl_2_), the culture was grown at 37 °C for 20 h with shaking at 250 rpm. The absorbance of the resulting cultures was measured at 600 nm using a spectrophotometer (MicroDigital Nabi).

### 2.8. Statistical Analysis

The data were analyzed by analysis of variance using IBM SPSS Statistics 23 software (IBM Corp., Armonk, NY, USA). Means with different letters indicate significantly different values at *p* < 0.05 according to Tukey’s post hoc honestly significant difference (HSD) test. Data are presented as means ± standard deviations.

## 3. Results

### 3.1. Gene Selection and Synthesis of the Escherichia coli RimI Gene

**Figure 1:**
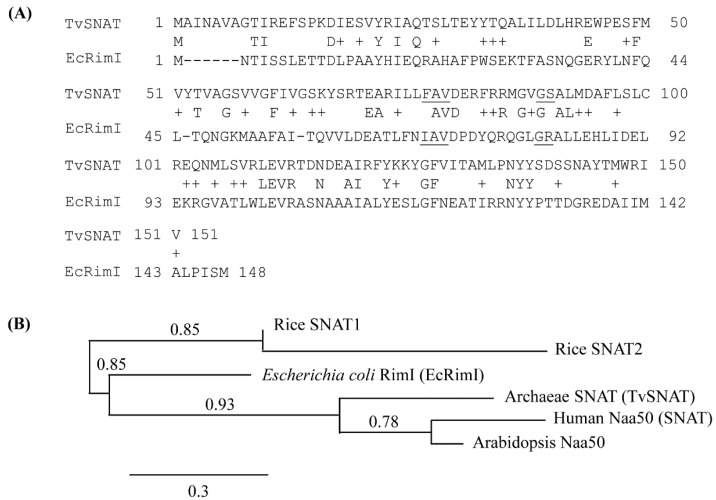
(**A**) Amino acid sequence alignment of TvSNAT and *E*. *coli* RimI (SNAT). The conserved acetyl coenzyme A binding sites are underlined. Plus signs (+) denote similar amino acids. Dashes denote gaps. (**B**) Phylogenetic tree showing *E*. *coli* SNAT, archaeal orthologues and rice SNAT genes. The scale bar represents 0.3 substitutions per site. GenBank accession numbers are: TvSNAT (NC_002689), *E*. *coli* RimI (WP_137442509), rice SNAT1 (AK059369), rice SNAT2 (AK068156), human Naa50 (BAB14397), and *Arabidopsis* Naa50 (NM_121172).

The BLASTp program (http://www.ncbi.nih.gov/, accessed on 3 November 2019) revealed that the archaeal SNAT (TvSNAT) protein [3] had ~23% identity and ~42% similarity to the RimI protein of *E*. *coli* (EcRimI), which encodes the protein *N*-acetyltransferase enzyme (Figure 1A). EcRimI acetylates a number of proteins, including ribosomal protein S18 [35], translation elongation factor Tu [36], and other proteins [37]. The human protein *N*-acetyltransferase Naa50, an orthologue of archaeal SNAT, shows SNAT enzyme activity [31], indicating that *E*. *coli* RimI was likely to also exhibit SNAT activity. Phylogenetic analysis indicated that EcRimI belonged to the SNAT family and was positioned between the plant and archaeal SNAT subfamilies, but much closer to the archaeal subfamily than the plant SNAT subfamily (Figure 1B). The full-length *EcRimI* nucleotide sequence was synthesized in accordance with sequence information reported in the GenBank database (WP_137442509).

### 3.2. Enzyme Kinetic Analysis of Recombinant EcRimI

**Figure 2:**
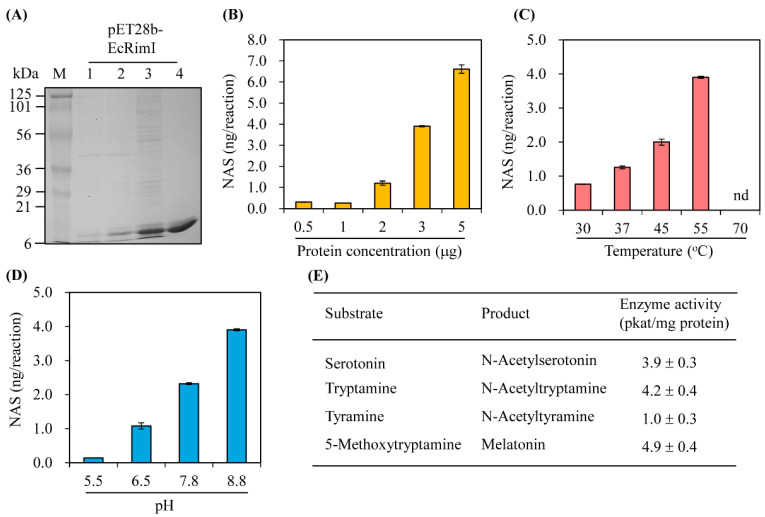
Affinity purification and enzymatic characteristics of *E. coli* RimI (EcRimI) protein. (**A**) Purification of C-terminal 6× His-tagged EcRimI protein. *E. coli* BL21 (DE3) cells harboring the pET28b-EcRimI plasmid were induced with isopropyl β-D-1-thiogalactopyranoside (IPTG) for 24 h at 24 °C. M, molecular mass standards; lane 1, total protein in 30 µL bacterial culture without IPTG; lane 2, total proteins in 30 µL bacterial culture with IPTG; lane 3, 10 µg soluble protein; lane 4, 5 µg protein purified through affinity chromatography. Protein samples were separated using 12% sodium dodecyl sulfate polyacrylamide gel electrophoresis (SDS-PAGE) and stained with Coomassie blue. Serotonin *N*-acetyltransferase enzyme activity as a function of (**B**) protein concentration, (**C**) temperature, (**D**) pH, and (**E**) substrate. Recombinant purified EcRimI (3 µg) was assayed for 0.5 h at 55 °C (varying temperature or pH) in the presence of 0.5 mM serotonin (or other substrate) and 0.5 mM acetyl coenzyme A, followed by high-performance liquid chromatography (HPLC) detection. NAS represents *N*-acetylserotonin. Values are means ± standard deviation (SD; *n* = 3).

**Figure 3:**
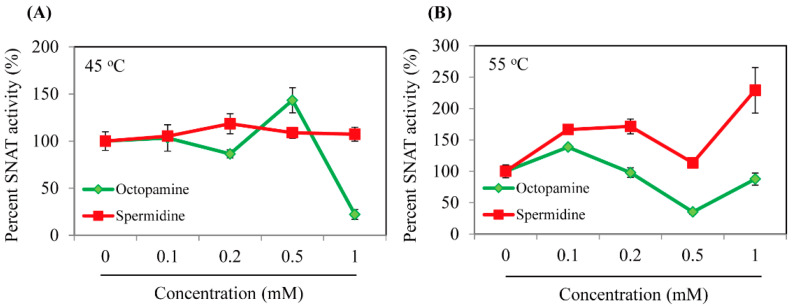
Dose-dependent inhibition of SNAT enzyme activity of recombinant EcRimI by polyamines. (**A**) SNAT enzyme inhibition in the presence of either spermidine or octopamine at 45 °C. (**B**) SNAT enzyme inhibition in the presence of either spermidine or octopamine at 55 °C. SNAT enzyme activity was measured in the presence of various levels of polyamines and 0.5 mM serotonin. SNAT activity is expressed as a percentage relative to that in the absence of polyamines. Values are means ± SD (*n* = 3).

The synthetic full-length *EcRimI* gene was cloned for expression as a fusion protein with a C-terminal hexa-histidine tag, followed by Ni^2+^ affinity purification, as illustrated in Figure 2A. The purified recombinant EcRimI protein was first investigated for the capacity to catalyze serotonin into *N*-acetylserotonin (NAS). As shown in Figure 2B, NAS was produced in vitro by recombinant EcRimI protein in a concentration-dependent manner. The optimal SNAT activity was observed at a temperature of 55 °C and half-peak activity occurred at 45 °C. SNAT activity at 37 °C was a quarter of that at 55 °C. No NAS was produced at 70 °C, which was consistent with archaeal SNAT [3]. EcRimI exhibited peak activity at pH 8.8, followed by pH 7.8 and pH 6.5, and very low SNAT activity was observed at pH 5.5. This pH preference of EcRimI SNAT activity was identical to that of archaeal SNAT, but differed from human Naa50, which has peak SNAT activity at pH 7.8 [3,31]. In addition to serotonin, several other amines were accepted as substrates. The maximum SNAT activity occurred with 5-methoxytryptamine, which was catalyzed into melatonin by the EcRimI enzyme, followed in rank order by tryptamine, serotonin, and tyramine. The substrate preference order of EcRimI differed from that of archaeal SNAT, in which tyramine is the optimal substrate and 5-methoxytryptamine is the least favorable substrate [3]. Tyramine has been reported as the optimal substrate for sheep SNAT and rice SNAT1 [38]. In terms of the possible acceptance of polyamines such as spermidine and octopamine as RimI substrates, we conducted an SNAT inhibition assay (0.5 mM serotonin) in the presence of each substrate at various concentrations to determine whether SNAT activity was affected by the presence of these polyamines. These inhibition analyses were performed because no standard compounds of *N*-acetylspermidine and *N*-acetyloctopamine are commercially available. As shown in Figure 3, spermidine did not inhibit SNAT activity at concentrations up to 1 mM under the 45 °C temperature condition. Similarly, octopamine slightly inhibited SNAT activity at 0.2 mM, but enhanced SNAT activity at 0.5 mM, shifting to 90% inhibition at 1 mM (Figure 3A). Under the 55 °C assay condition, spermidine enhanced SNAT activity, whereas octopamine did not alter SNAT activity except at 0.5 mM, where it showed a 50% inhibition rate (Figure 3B). Collectively, *E*. *coli* SNAT (EcSNAT or EcRimI) likely did not accept polyamines as substrates, in sharp contrast to archaeal SNAT, which accepts polyamines as substrates [3].

**Figure 4:**
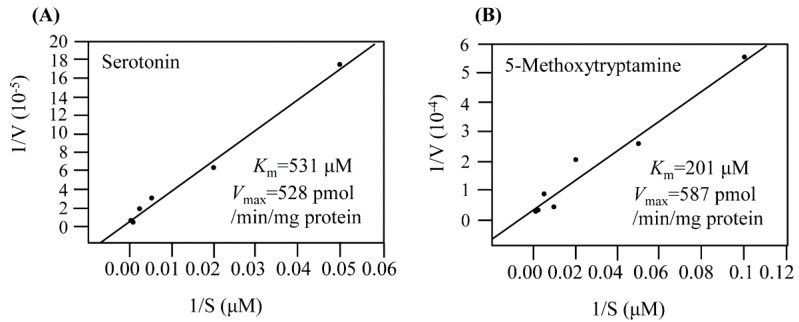
Determination of *K*_m_ and *V*_max_ values for recombinant EcRimI toward the substrates (**A**) serotonin and (**B**) 5-methoxytryptamine. *K*_m_ and *V*_max_ values were determined using Lineweaver–Burk plots at 55 °C.

The *K*_m_ and *V*_max_ values of EcRimI toward serotonin as a substrate were 531 μM and 528 pmol/min/mg protein, respectively (Figure 4A). For the substrate 5-methoxytryptamine, EcRimI exhibited *K*_m_ and *V*_max_ values of 201 μM and 587 pmol/min/mg protein, respectively (Figure 4B). The catalytic efficiency (*V*_max_/*K*_m_) was 3-fold higher toward 5-methoxytryptamine than toward serotonin, suggesting that EcRimI preferred 5-methoxytryptamine to serotonin as a substrate for melatonin biosynthesis. The enzyme kinetics values of EcRimI were very similar to those of archaeal SNAT, supporting EcRimI as an archaeal SNAT orthologue. By contrast, human Naa50, a known archaeal SNAT orthologue, exhibited much higher *K*_m_ and *V*_max_ values for serotonin of 986 μM and 1800 pmol/min/mg protein, respectively. Based on the data presented above regarding the enzymatic properties of SNAT, EcRimI possessed SNAT activity, in accordance with archaeal SNAT and human Naa50 [3,31]. However, the exact mechanism through which EcRimI synthesizes melatonin in *E*. *coli* awaits further in-depth analysis, particularly in view of the dual roles of EcRimI in melatonin synthesis and protein *N*-acetylation.

### 3.3. Escherichia coli Growth Curves for the EcRimI Overexpression Strain

**Figure 5:**
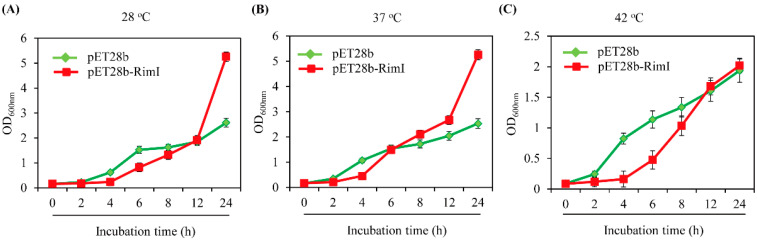
Growth curves of control (pET28b) and *EcRimI* overexpression (pET28b-RimI) *E*. *coli* strains at (**A**) 28 °C, (**B**) 37 °C, and (**C**) 42 °C. Each point represents three independent replicates. *E*. *coli* was grown in nutrient-rich TB medium.

The *E*. *coli rimI* mutant strain exhibits moderate growth retardation in minimal medium, but this growth difference is nonsignificant in nutrient-rich medium such as LB medium [36]. Importantly, as temperature strongly affects the solubility of the recombinant protein expressed in *E*. *coli* [39], we tested a wide range of temperatures for comparison of *E*. *coli* growth between the control (pET28b) and overexpression (pET28b-RimI) strains. In contrast to the previous study with the *rimI* mutant strain, we used nutrient-rich media such as Terrific broth (TB) medium to examine the growth effects of *EcRimI* overexpression. A significant growth disadvantage of the *EcRimI* overexpression strain relative to the control strain was apparent during the first 8 h of culture, but this growth retardation of the *EcRimI* overexpression strain was overcome at 12 h, and 2-fold higher growth was achieved at 24 h under the 28 °C culture temperature condition (Figure 5A). The growth recovery effect in the *EcRimI* overexpression strain was faster and more pronounced at the 37 °C culture temperature than at 28 °C. The *EcRimI* overexpression strain grew more slowly than the control during the first 4 h incubation and reached an equal growth rate comparable to the control strain at 6 h, then grew faster than the control strain from 8 h, and finally showed more than 2-fold higher growth than the control strain at 24 h (Figure 5B). At the 42 °C culture temperature, a similar growth disadvantage of the *EcRimI* overexpression strain was observed until 8 h, and OD_600_ values equal to those of the control strain were achieved at 12 h. At the end of 24 h of culturing at 42 °C, the growth advantage of *EcRimI* overexpression was marginal compared to that at 28 and 37 °C (Figure 5C). These data clearly indicated that *EcRimI* overexpression conferred a strong ability to enhance *E*. *coli* growth at the later stage of bacterial cultivation compared to the control strain, although whether these positive effects resulted from either protein *N*-acetylation or melatonin synthesis remained unclear. This mechanism should be investigated in the near future.

### 3.4. Melatonin Production in Escherichia coli

**Figure 6:**
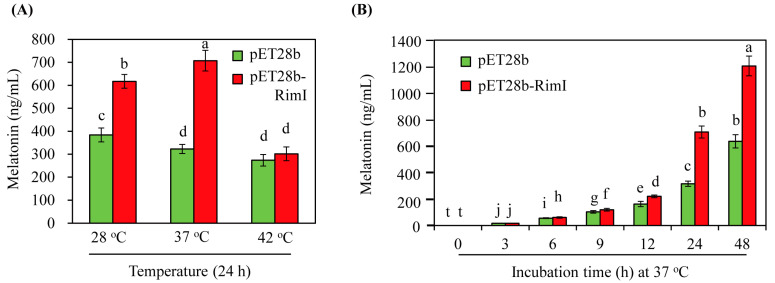
(**A**) Melatonin production of the control (pET28b) and *EcRimI* overexpression (pET28b-RimI) strains at 24 h under various culture temperature conditions. (**B**) Time course of melatonin production by the control (pET28b) and *EcRimI* overexpression (pET28b-RimI) strains at 37 °C. Medium fractions were subjected to HPLC analysis for melatonin quantification. Different letters indicate significant differences from the wild type (Tukey’s honest significant difference (HSD) test; *p* < 0.05). Values are presented as means ± SD (*n* = 3).

*E*. *coli* produces small amounts of melatonin (>1 ng/mL) [34]. To identify melatonin in *E*. *coli*, 5-methoxytryptamine, a direct substrate of melatonin production by SNAT, was added to the culture medium. After 24 h incubation in the presence of 1 mM 5-methoxytryptamine and 1 mM isopropyl β-D-1-thiogalactopyranoside (IPTG) at various temperatures, melatonin levels were quantified in the medium fraction of the *E*. *coli* culture, as the majority of melatonin was found in the medium fraction rather than in *E*. *coli* cells [34]. As shown in Figure 6A, the control *E*. *coli* strain (pET28b) also produced melatonin at concentrations of 383, 322, and 301 ng/mL at the incubation temperatures of 28, 37, and 42 °C, respectively. By contrast, the *EcRimI* overexpression *E*. *coli* strain (pET28b-RimI) produced melatonin at 617, 707, and 301 ng/mL at 28, 37, and 42 °C, respectively. Thus, the *EcRimI* overexpression strain produced melatonin at rates 1.6- and 2.2-fold higher than the control strain when *E*. *coli* was incubated at 28 and 37 °C, respectively. However, no differences in melatonin production between the *EcRimI* overexpression and control strains were observed at 42 °C culture temperature. These results were consistent with the growth curves at 42 °C. Thus, the optimal temperature for melatonin production in the *EcRimI* overexpression *E*. *coli* strain was 37 °C, followed by 28 °C. Time-course analysis of melatonin production was performed at the optimal temperature of 37 °C (Figure 6B). Melatonin production increased as the incubation time was prolonged. However, in the absence of 5-methoxytryptamine, the melatonin level was below our HPLC detection limit of 1 ng/mL. Our data suggested that *E*. *coli* could synthesize melatonin in the presence of 5-methoxytryptamine, which is present in the diet [40,41]. A biosynthetic pathway from serotonin to *N*-acetylserotonin and then to melatonin is also possible, but is unlikely due to the requirement of an additional enzyme, such as ASMT. These possibilities remain to be investigated in future research.

### 3.5. Cadmium Response of the EcRimI Overexpression Strain

**Figure 7:**
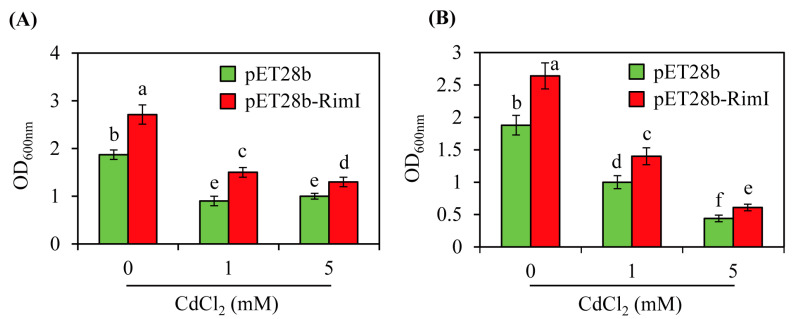
Growth curves of the control (pET28b) and *EcRimI* overexpression (pET28b-RimI) *E*. *coli* strains in the presence of cadmium. (**A**) Cadmium was applied to a culture at OD_600_ = 1.0 and incubated for 20 h at 37 °C. (**B**) Cadmium was applied to a culture at OD_600_ = 0.5 and incubated for 20 h at 37 °C. The medium used in this experiment was LB (Lennox broth). Overnight seed cultures of 100 µL were inoculated into 1 mL fresh LB medium containing the antibiotic kanamycin (50 µg/mL) and grown at 37 °C until reaching OD_600_ = 1.0 or 0.5, followed by cadmium treatment for 20 h. Each point represents three independent replicates. Different letters indicate significant differences from the wild type (Tukey’s HSD test; *p* < 0.05).

Cadmium treatment of *E*. *coli* inhibits antioxidant enzymes and lowers the levels of antioxidants such as glutathione, which decreases the scavenging of reactive oxygen species and leads to mechanical damage [42]. Correspondingly, adding glutathione alleviates damage from oxidative stress and increases the growth rate of *E*. *coli* [43]. In contrast to glutathione, melatonin treatment hampers the growth of *Xanthomonas oryzae*, a plant-pathogenic bacterium [24]. To assess the impacts of cadmium stress, the *EcRimI* overexpression strain was challenged with cadmium treatment. As shown in Figure 7, the *EcRimI* overexpression strain did not exhibit cadmium stress tolerance compared to the control. However, even in the presence of cadmium, the *EcRimI* overexpression strain of *E*. *coli* showed significantly enhanced growth relative to the control strain, regardless of the cell density. These data suggested that the growth enhancement associated with *EcRimI* overexpression was barely affected by heavy metal stress, indicating that EcRimI played a general role in improving *E*. *coli* growth through modulation of protein *N*-acetylation, melatonin biosynthesis, or both.

## 4. Discussion

The biosynthetic pathways and biological roles of melatonin have been well documented since its discovery in the pineal gland of cows in 1958 [44] and in several plant species in 1995 [45,46]. Accordingly, increasing numbers of melatonin biosynthesis-related genes have been cloned and characterized from prokaryotes and eukaryotes through sequence homology-based cloning strategies using sheep SNAT [27,47]. Among such genes, *SNAT* has been most widely studied, as it plays a key role in melatonin biosynthesis in diverse organisms [48]. Consequently, many sheep *SNAT* orthologues have been cloned from human [49], yeast [50], *Drosophila melanogaster* [51], *Chlamydomonas reinhardtii* [52], and *Xanthomonas oryzae* [53] (Table 1). Similarly, plant *SNAT* genes have been cloned from various species [54]. In contrast to the single copy of *SNAT* present in animals [28], plant *SNAT* exists as a gene family containing at least 3 isogenes with very low amino acid sequence homology [25]. These *SNAT* genes include rice *SNAT1* [29,55] and *SNAT2* [38], *Arabidopsis SNAT1* [56] and *SNAT2* [11], tobacco *SNAT1* and *SNAT2* [57], apple *SNAT3* [58], red algal *SNAT* [59], and cyanobacterial *SNAT* [30]. All plant SNAT1 and SNAT2 expression is localized in chloroplasts, except apple SNAT3, which is expressed in mitochondria [58]. A mitochondrial apple SNAT3 orthologue has been found in rice, but its function as an SNAT enzyme in rice remains unknown [25]. Collectively, rice likely contains up to four *SNAT* isogenes including an archaeal orthologue, which is expressed in the cytoplasm, in accordance with human Naa50, another archaeal *SNAT* orthologue [31]. The archaeal *SNAT* gene was recently cloned and characterized from archaeal GNAT family genes [3]. This archaeal *SNAT* from *Thermoplasma volcanium* was previously annotated as TvArd1 (arrest-defective-1), encoding a protein with *N*-terminal acetyltransferase activity that transfers an acetyl group from acetyl-coenzyme A to the *N*-termini of various proteins [60,61,62]. Ard1 is also called Naa10, and it is one of six NAT enzyme complexes containing Nat10 to Nat60 [63]. The closest orthologue of archaeal SNAT in humans is Naa50, *N*-alpha-acetyltransferase 50, which was recently revealed to possess SNAT enzyme activity [31]. These data suggest that archaeal SNAT proteins with *N*-acetyltransferase activity represent a novel class of *SNAT* family genes. In support of this possibility, *E*. *coli RimI*, an archaeal *SNAT* orthologue that exhibits protein *N*-acetyltransferase activity, also showed SNAT enzyme activity, indicating that *E*. *coli* have the genetic capacity to synthesize melatonin from its penultimate precursor, serotonin, as well as the direct substrate 5-methoxytryptamine (Figure 2). Phylogenetic analysis indicated that EcRimI belongs to the archaea SNAT family comprising TvSNAT [3] and human Naa50 [31] and is distantly related to the animal SNAT and plant SNAT family, suggesting that EcRimI is a functional *E. coli* orthologue of archaeal SNAT. In addition to the *SNAT* orthologue genes found in animals, plants, and archaea, enhanced intracellular survival (Eis) proteins containing two NAT domains and one sterol carrier domain also exhibit SNAT enzyme activity; however, Eis homolog proteins are present mainly in mycobacteria and certain Gram-positive bacteria, but are not found in animals and plants [64].

The different activities of SNAT enzymes derived from plants and archaea have triggered fundamental questions about their biological functions, such as whether transgenic phenotypes showing gain or loss of *SNAT* gene function can be attributed to changes in melatonin or protein acetylation. *Arabidopsis SNAT1* exhibits *N*-acetyltransferase activity toward chloroplast proteins [65], and its knockout mutant of *SNAT1* (*snat1*) exhibited stunted growth depending on light intensity [12]. Whether the main reason for altered phenotypes in the *snat1* mutant of *Arabidopsis* is decreased biosynthesis of melatonin, altered levels of chloroplast protein acetylation, or the combined effects of both changes remains unclear [12,20,65]. Analogously, *Naa50* knockdown disrupted centromeric sister chromatid cohesion in *Drosophila* [66] and centromeric cohesion in HeLa cells [67], suggesting that protein *N*-acetyltransferase activity plays an important role in cell division. However, these findings do not eliminate the possible involvement of melatonin in cell division. This possibility remains to be elucidated in future research. Notably, the phenotypic abnormality observed in humans and *Drosophila* was not found in *Naa50* knockdown yeast [68]. *E*. *coli* RimI is responsible for the *N*-terminal acetylation of the ribosomal protein S18 [69] and elongation factor Tu [36]. Consequently, an *E*. *coli* strain devoid of *RimI* showed slightly reduced growth on minimal medium, which was not observed in nutrient-rich medium, possibly driven by reduced efficiency of translation [36]. Assessing whether the growth retardation of the *RimI*-lacking strain is associated with melatonin biosynthesis remains an intriguing question.

All *SNAT* genes belong to the GCN5-related *N*-acetyltransferase (GNAT) superfamily of enzymes and share a common acetyl coenzyme A binding domain [70]. Although whether animal SNAT proteins such as sheep SNAT have protein *N*-acetyltransferase activity remains unclear, SNAT proteins derived from plants and archaea exhibit such activity [36,65]. The dual activity of SNAT proteins gives rise to the production of melatonin, *N*-acetylated proteins, or both simultaneously. Both products play diverse biological roles in organisms. Melatonin is a universal and pleiotropic molecule orchestrating the day–night waking cycle, seasonal reproduction, antitumor functions, and immune responses in animals [2,71] while acting as a master regulator of plant growth and development [72]. *N*-acetylation is a universal protein modification process, with 80–90% of soluble proteins being *N*-terminally acetylated [62], and regulates protein degradation, subcellular translocation, and protein complex formation [63].

As reported in animals and plants, melatonin may have certain biological functions, such as antioxidation, in *E*. *coli* [4,41,73,74]. The *EcRimI* mutant strain showed no growth inhibition in nutrient-rich LB medium [37]. In addition, the *EcRimI* overexpression strain exhibited far less *N*-acetylation activity toward *E*. *coli* proteins in vivo than other *N*-acetyltransferases such as YfiQ, Yjab, and YiaC, suggesting that the role of EcRimI as a protein N-acetyltransferase is minimal [37]. Although no direct evidence connects melatonin with *E*. *coli* growth, a beneficial effect of melatonin on *E*. *coli* growth cannot be ruled out, as the *EcRimI* overexpression *E*. *coli* strain showed enhanced growth in conjunction with increased melatonin production in this study. Melatonin has long been proposed to be synthesized in bacteria, especially in *E. coli*, but no biosynthesis pathway has been found. Due to the discovery *SNAT* gene in *E. coli*, we open a new window to studying the function of melatonin in bacteria by way of gene manipulation and to engineer melatonin biosynthesis for its overproduction in *E. coli*. This is the first report of successful cloning of *SNAT* from *E*. *coli*. Further studies will shed light on the physiological roles of melatonin in *E*. *coli* during growth and under oxidative stress.

## 5. Conclusions

With the help of the first successful cloning of archaeal *SNAT*, we revealed that *E*. *coli RimI*, encoding a protein *N*-acetyltransferase, exhibited SNAT enzyme activity in vitro and that *EcRimI* overexpression in vivo was associated with enhanced melatonin production in *E*. *coli*. The *EcRimI* overexpression *E*. *coli* strain exhibited enhanced growth compared to the control strain. The *EcRimI* overexpression *E*. *coli* strain showed strong tolerance against stationary-phase stress based on its growth curves. This enhanced growth effect of *EcRimI* overexpression compared to that of the control remained in the presence of cadmium stress. In summary, *E*. *coli* clearly has the capacity to synthesize melatonin through the enzymatic activity of EcRimI using 5-methoxytryptamine present in the diet, and *EcRimI* overexpression plays an important role in *E*. *coli* growth, possibly associated with melatonin synthesis, protein acetylation, or both.

## Figures and Tables

**Table 1:** Enzyme kinetics of SNAT proteins from various organism.

Organism	Enzyme	*K*_m_ (μM)		*V*_max_		Reference
		Serotonin	nmol/min/mg Protein			
Animal SNAT orthologue proteins						
Human	SNAT	1235	-			[49]
Sheep	SNAT	85	0.67			[46]
Yeast	SNAT	5100	-			[50]
*Drosophila melanogaster*	SNAT	1620	-			[51]
*Xanthomonas oryzae*	SNAT	709	-			[53]
*Chlamydomonas reinhardtii*	SNAT	247	0.325			[52]
Plant SNAT orthologue proteins						
Rice	SNAT1	270	3.3			[55]
Rice	SNAT2	371	4.7			[38]
Arabidopsis	SNAT1	309	1.4			[56]
Arabidopsis	SNAT2	232	2.1			[11]
Tobacco	SNAT1	579	8.1			[57]
Tobacco	SNAT2	326	1.5			[57]
Apple	SNAT3	55	0.0009			[58]
Red algae	SNAT	467	28			[59]
Cyanobacteria	SNAT	823	1.6			[30]
Archaea SNAT orthologue proteins						
*Thermoplasma volcanium*	SNAT	621	0.416			[3]
Human	SNAT	986	1.8			[31]
*Escherichia coli*	SNAT	531	0.528			This paper
Enhanced intracellular survival (Eis) protein						
*Saccharopolyspora erythraea*	SNAT	13,000	-			[64]

## Data Availability

The data that support the finding of this study are available from the corresponding author upon reasonable request.
